# Rapid Physiological Fluctuations in Nucleus Accumbens Oxygen Levels Induced by Arousing Stimuli: Relationships with Changes in Brain Glucose and Metabolic Neural Activation

**DOI:** 10.3389/fnint.2017.00009

**Published:** 2017-04-24

**Authors:** Ernesto Solis Jr., Keaton T. Cameron-Burr, Eugene A. Kiyatkin

**Affiliations:** In-Vivo Electrophysiology Unit, Behavioral Neuroscience Branch, National Institute on Drug Abuse—Intramural Research Program, Department of Health and Human Services, National Institutes of HealthBaltimore, MD, USA

**Keywords:** arousing stimuli, neural activation, neuro-vascular coupling, oxygen electrochemistry, respiration, rats

## Abstract

Proper entry of oxygen from arterial blood into the brain is essential for maintaining brain metabolism under normal conditions and during functional neural activation. However, little is known about physiological fluctuations in brain oxygen and their underlying mechanisms. To address this issue, we employed high-speed amperometry with platinum oxygen sensors in freely moving male rats. Recordings were conducted in the nucleus accumbens (NAc), a critical structure for sensorimotor integration. Rats were exposed to arousing stimuli of different nature (brief auditory tone, a 1-min novel object presentation, a 3-min social interaction with a conspecific, and a 3-min tail-pinch). We found that all arousing stimuli increased NAc oxygen levels. Increases were rapid (4–10-s onset latencies), modest in magnitude (1–3 μM or 5%–15% over baseline) and duration (5–20 min), and generally correlated with the arousing potential of each stimulus. Two strategies were used to determine the mechanisms underlying the observed increases in NAc oxygen levels. First, we showed that NAc oxygen levels phasically increase following intra-NAc microinjections of glutamate (GLU) that excite accumbal neurons. Therefore, local neural activation with subsequent local vasodilation is involved in mediating physiological increases in NAc oxygen induced by arousing stimuli. Second, by employing oxygen monitoring in the subcutaneous space, a highly-vascularized area with no metabolic activity, we determined that physiological increases in NAc oxygen also depend on the rise in blood oxygen levels caused by respiratory activation. Due to the co-existence of different mechanisms governing oxygen entry into brain tissue, NAc oxygen responses differ from fluctuations in NAc glucose, which, within a normal behavioral continuum, are regulated exclusively by neuro-vascular coupling due to glucose’s highly stable levels in the blood. Finally, we discuss the relationships between physiological fluctuations in NAc oxygen, glucose and metabolic brain activation assessed by intra-brain heat production.

## Introduction

The brain is one of the heaviest consumers of oxygen in the body and accounts for ~20% of total oxygen consumption (Siesjo, 1978; Rolfe and Brown, 1997). The high metabolic activity of the central neurons requires constant and efficient delivery of oxygen, which enters the brain extracellular space from the arterial blood via gradient-dependent diffusion (Attwell et al., 2010). Oxygen entry into the brain is enhanced during functional neural activation and it is generally believed that this adaptive effect results from neuronal activation that induces local vasodilation and increases local cerebral blood flow (CBF; Lecrux and Hamel, 2011; Muoio et al., 2014). It is well known that neural activation results in enhanced oxygen consumption (Siesjo, 1978), implying possible decreases in extracellular oxygen levels following increased neural activity. Neural activation is also accompanied by rapid increases in local CBF (Fox and Raichle, 1986; Fellows and Boutelle, 1993; Martin et al., 2006; Paulson et al., 2010), suggesting enhanced delivery of oxygen into the brain tissue. In addition, due to the gradient dependent nature of oxygen entry via the blood-brain barrier, oxygen levels in brain tissue also depend on the degree of oxygenation of arterial blood. This parameter is determined by respiration, which is highly variable and affected by multiple factors including animal activity state and ongoing behavior. Given these three dynamic and potentially opposing influences, it remains unclear how the balance between oxygen consumption and its entry from arterial blood is maintained under physiologically relevant conditions and how it is changed during neural activation elicited by natural arousing stimuli.

To address this issue, we used newly developed oxygen sensors (Pinnacle Technology, Inc., Lawrence, KS, USA) coupled with fixed-potential amperometry in awake, freely moving rats to examine physiological fluctuations in extracellular oxygen levels in the nucleus accumbens (NAc), a critical structure for sensorimotor integration and a key component of the brain motivation-reinforcement circuit (Mogenson et al., 1980; Wise and Bozarth, 1987). Although the principles of voltammetric oxygen detection in brain tissue have a long history (Davies and Brink, 1942; Davies and Bronk, 1957; Clark et al., 1958) and the development of small oxygen sensors coupled with high-speed amperometry (Lowry et al., 1997; Bolger and Lowry, 2005; Bolger et al., 2011; Francois et al., 2012; Kealy et al., 2013; Lyons et al., 2016) or cyclic voltammetry (Wang and Venton, 2016) have made it possible to evaluate changes in oxygen levels in discrete brain areas with high temporal resolution, data on physiological fluctuations in brain oxygen levels are very limited.

This report presents three sets of original data. First, we examined changes in NAc oxygen levels induced by arousing stimuli of different nature and salience (auditory stimulus, novelty test, tail-pinch, and social interaction). A brief auditory tone is a weak sensory stimulus that induces transient cortical EEG desynchronization (McClung et al., 1976–77; Sasaki et al., 1996; Kiyatkin and Smirnov, 2010) without evident behavioral effects. In contrast, exposure to a novel object, tail-pinch and social interaction with a conspecific individual are complex arousing stimuli that induce relatively strong and prolonged behavioral and physiological responses (Kiyatkin, 2010). In addition, we conducted two experiments to elucidate the mechanisms that may control physiological fluctuations in NAc oxygen levels. To directly assess the role of neuronal activation in mediating rapid fluctuations in NAc oxygen, we examined how oxygen levels in this brain structure are affected following direct neuronal activation induced by local microinjections of glutamate (GLU) near the oxygen detection site. Since entry of oxygen into the brain tissue directly depends on its levels in arterial blood, we also conducted oxygen measurements in the subcutaneous site, a densely-vascularized area with no metabolic activity. The subcutaneous location was previously used as an effective proxy for assessing blood glucose levels (Moon et al., 2013); glucose values directly recorded from the arterial blood and subcutaneous location were virtually identical. Recordings conducted at this location provide a reliable measure of tissue oxygen that is independent of tissue oxygen consumption.

Although our primary goal was to detect physiological fluctuations in brain oxygen and explore their possible mechanisms, two more general issues were also considered. First, we examined how oxygen is related to glucose, another critical substrate for maintaining metabolic brain activity, which, similarly to oxygen, enters the brain tissue via a gradient-dependent mechanism (Siesjo, 1978; Sokoloff, 1999; Mergenthaler et al., 2013). The present data on fluctuations in NAc oxygen were compared with our previous results obtained with enzyme-based glucose biosensors using the same experimental protocol (Kiyatkin and Lenoir, 2012; Kiyatkin and Wakabayashi, 2015). We also explored the relationship between changes in oxygen and metabolic brain activation, using the results of our previous brain thermorecording studies (Kiyatkin et al., 2002; Kiyatkin, 2010). Although intra-brain entry of both oxygen and glucose are usually considered as a response to metabolic demand or increased metabolism (Duelli and Kuschinsky, 2001; Masamoto and Tanishita, 2009; Muoio et al., 2014), most assessments of brain metabolism are indirect and have relied on changes in neuronal activity, CBF, or a central vascular response—rapid neural effects that precede much slower changes in metabolism (Ritchie, 1973; Sokoloff, 1999). Brain metabolism can be more directly characterized by oxygen consumption and/or glucose utilization (Sokoloff, 1999), but these parameters are discrete and do not allow dynamic monitoring. Since cerebral metabolism is accompanied by heat production (Hodgkin, 1967; Ritchie, 1973; Siesjo, 1978), brain temperature monitoring can provide reliable information on dynamic changes in brain metabolic activity.

## Materials and Methods

### Subjects and Surgical Preparations

Fourteen adult male Long-Evans rats (Charles River Laboratories, Wilmington, MA, USA) weighing 460 ± 40 g at the time of surgery were used in this study. Rats were individually housed in a climate-controlled animal colony maintained on a 12–12 light-dark cycle (lights on at 6:00), with food and water available *ad libitum*. All procedures were approved by the NIDA-IRP Animal Care and Use Committee and complied with the Guide for the Care and Use of Laboratory Animals (NIH, Publication 865-23). Maximal care was taken to minimize the number of experimental animals and any possible discomfort or suffering at all stages of the study.

This study describes the results of three electrochemical experiments. In Experiment I (11 recording sessions in seven rats), we examined changes in NAc oxygen levels induced by natural arousing stimuli. In Experiment II (five recording sessions in four rats), we examined changes in NAc oxygen levels induced by microinjection of GLU near the oxygen sensing area. In Experiment III (three recording sessions in three rats), we examined how select arousing stimuli affect oxygen levels in the subcutaneous site.

Surgical procedures for electrochemical experiments were identical in all experiments and were described in detail elsewhere (Kiyatkin and Lenoir, 2012). Under general anesthesia (Equithesin, a mixture of sodium pentobarbital and chloral hydrate), each rat was unilaterally implanted with a BASi guide cannula (Bioanalytical Systems, West Lafayette, IN, USA) into which an electrochemical sensor was later inserted. In Experiment I, cannulae were implanted for recordings in the right NAc shell. Target coordinates of the recordings were: AP +1.2 mm, ML ±0.8 mm, and DV +7.6 mm from the skull surface, according to coordinates of the rat brain atlas of Paxinos and Watson (1998). In Experiment II, rats were implanted with a modified cannula connected in parallel with a small diameter stainless steel needle that was used for GLU delivery into the brain tissue near the sensor’s sensing area. This modified cannula was used previously to examine the role of neural activity in mediating glucose entry into the brain tissue (Kiyatkin and Lenoir, 2012). In Experiment III, cannulae were implanted subcutaneously in the medio-frontal area of the rat’s head. The guide cannula in each experiment was secured with dental acrylic in a head mount anchored to the skull. When not in use, stainless steel obdurators were inserted into the cannulae to prevent occlusions. Rats were allowed a minimum of 5 days of post-operative recovery and at least 3 daily habituation sessions (~6 h each) to the recording environment; jugular catheters were flushed daily with 0.2 ml heparinized saline to maintain patency.

### Oxygen Sensors and Electrochemical Detection of Oxygen in Brain Tissue

In our experiments, we used commercially produced oxygen sensors (Model 7002-02; Pinnacle Technology, Inc., Lawrence, KS, USA). These sensors consist of epoxy-sheathed disc electrodes that are grounded to a fine surface using a diamond-lapping disc. These sensors are prepared from Pt-Ir wire 180 μm in diameter, with a sensing area of 0.025 mm^2^ at the sensor’s tip. The active electrode is incorporated with an integrated Ag/AgCl reference electrode. On the active surface of these sensors, which is held at a stable potential of −0.6 V vs. the reference electrode, dissolved oxygen is reduced producing an amperometric current. The current from the sensor is passed to a computer via a potentiostat (Model 3104, Pinnacle Technology, Inc., Lawrence, KS, USA) and recorded at 1-s intervals, using the PAL software utility (Version 1.5.0, Pinnacle Technology Inc., Lawrence, KS, USA).

In this study, we used two types of oxygen sensors (polyphenol-coated and bare), which have minor differences in *in vitro* oxygen sensitivity and in *in vivo* performance (see “Results” Section). Calibration of oxygen sensors was performed by the manufacturer (Pinnacle Technology Inc., Lawrence, KS, USA) according to a standard protocol described elsewhere (Bolger et al., 2011). Calibrations were conducted in a stirred solution of 100 mM PBS buffer at 37°C. Oxygen was purged from the testing solution by bubbling argon through the solution for at least 20 min. An oxygen-saturated solution was made by bubbling oxygen through a separate solution of 100 mM PBS buffer. After 20 min of argon bubbling the tube supplying argon was removed from the buffer solution and placed above the testing solution to maintain an argon atmosphere. Oxygen sensors were then calibrated by multiple injections of the oxygen saturated (1.2 mM) solution into the oxygen-purged testing solution. Sensors used in this study produced an incremental current rise with increases in oxygen concentrations within the wide range of previously reported brain oxygen concentrations (0–50 μM). Substrate sensitivity of oxygen sensors varied from 1.0 to 1.8 nA/1 μM (mean = 1.35 nA/1 μM; SD = 0.27 nA). Oxygen sensors were also tested by the manufactures for their selectivity toward other electroactive substances, including dopamine (0.4 μM) and ascorbate (250 μM), which had no significant effects on reduction currents.

### Experimental Procedures

All *in vivo* electrochemical procedures were performed in an electrically insulated chamber (38 × 47 × 47 cm) located in a larger open-faced cabinet. The cage was illuminated continuously with a dim 20 W light bulb; a room wide air filter fan provided background noise and ambient temperature was maintained within 22–23°C. The bottom of the cage was covered with wood chip bedding, which remained in place during the habituation and recording sessions for each individual rat. Prior to recording, rats were habituated to the testing environment for a minimum of 6 h per day for three consecutive days.

At the beginning of each experimental session, rats were minimally anesthetized (<2 min) with isoflurane and the sensor was inserted into the NAc through the guide cannula. The rat was then placed in the testing chamber and the sensor connected to the potentiostat via an electrically shielded flexible cable and a multi-channel electrical swivel. Testing began a minimum of 120 min after insertion of the sensor when the baseline current stabilized. In Experiment I, rats were exposed to four types of physiological stimuli: a brief auditory stimulus (0.5-s, 75 dB), novel object presentation (a glass beaker manually placed in the recording chamber and removed 60 s later), tail-pinch, and social interaction. A wooden clothespin was manually attached to the base of the tail during the tail-pinch stimulus and removed 3 min later. In contrast to other studies that employed a metal clamp, this is a mild arousing stimulus that is neither painful nor harmful to the animal. For the social interaction, a novel male conspecific of similar age and weight was introduced into the cage for 3 min. Time intervals between stimulus presentations were 40–60 min, typically enough for restoration of pre-stimulus current baselines.

In Experiment II, we used a modified cannula design, which was previously employed to examine the role of neural activity in regulating glucose entry into brain tissue (Kiyatkin and Lenoir, 2012). Briefly, a BASi guide cannula was glued in parallel with a small diameter stainless steel needle (28G, external diameter 360 μm, internal diameter 120 μm, internal volume ~0.5 μl) that allowed microinjections of GLU into the brain tissue near the sensor’s sensing area (~300–400 μm). On the day of an experiment, under short-term isoflurane anesthesia, an oxygen sensor was inserted into the cannula and the top of the intracranial needle was connected to a catheter extension filled with a 5 mM solution of GLU (L-glutamate monosodium salt) and secured to the electrical cable. This catheter extension was then connected via tubing to a pump (Model A-99; Razel Scientific Instruments, Inc., St. Albans, VT, USA) with an injection syringe (Hamilton Co, Reno, ND, USA) that allowed slow delivery of the solution during electrochemical recordings. Since the injection needle was pre-filled with a neutral solvent, the design of this parallel sensor cannula-intracranial injection device allowed us first to test the effects of a control vehicle injection (first 1–3 microinjections) and then to examine the effects of GLU. As shown previously, dorsal and ventral striatal neurons in awake, unrestrained rats are very sensitive to local applications of GLU, eliciting dose-dependent excitations (Kiyatkin and Rebec, 1999). GLU was injected several times during each recording session (*n* = 4–8; 0.5–4.0 μl) and the GLU dose was adjusted by the duration of injection.

In Experiment III, in which oxygen recordings were conducted in the subcutaneous site, we used a similar protocol, focusing on two arousing stimuli that induced distinct NAc responses in Experiment I. At the end of each session, rats were anesthetized with Equithesin, disconnected from the potentiostat, and the biosensor was carefully removed. Typically, rats underwent only one recording session and upon completion they were euthanized by decapitation under deep isoflurane anesthesia. Then, the brain was removed, stored in 10% formalin, and later sectioned for verification of sensor placement using the stereotaxic atlas of Paxinos and Watson (1998) and assessed for possible damage of brain tissue. Electrochemical data were accepted only if the sensor tips were localized in the NAc shell and the tissue had minimal damage. In several rats, electrochemical recordings were repeated 2–4 days later.

### Data Analysis

Electrochemical data were sampled at 1 Hz (i.e., mean current over 1 s) using the PAL software utility (Version 1.5.0, Pinnacle Technology Inc., Lawrence, KS, USA) and analyzed with two time resolutions. Slow changes in electrochemical current were analyzed with 60-s quantification bins for 3 min before and 10 (sound), 20 (novel object presentation), and 40 min (tail-pinch and social interaction) after stimulus onset. Rapid current changes were analyzed with 4-s bins for 40 s before and 300 s after stimulus onset. Although data were sampled with 1-s temporal resolution, the 4-s bin appears to be optimal for detecting rapid current changes within the time scale of our stimuli while simultaneously reducing the contribution of electric noise.

Electrochemical data were first analyzed in terms of currents. Because our sensors differed slightly in background currents and substrate sensitivity *in vitro*, the currents were transformed into concentrations and represented as relative concentration changes, taking a pre-stimulus baseline current as 0. We also determined the rate of oxygen concentration change by calculating the difference in concentration between subsequent values and representing it as a function of time. Basal oxygen currents and their concentration equivalents were also used to estimate basal levels of oxygen in the NAc and subcutaneous space. One-way repeated measure analysis of variances (ANOVAs; followed by Fisher LSD *post hoc* tests) were used to evaluate the statistical significance of stimulus-induced changes in oxygen concentrations. The latency of oxygen response was determined based on the first data point significantly different from baseline (*p* < 0.05, Fisher test). We also used correlation analyses to examine the relationship between oxygen, glucose, and several temperature parameters.

## Results

### Basal Extracellular Oxygen Levels in the Nucleus Accumbens

Since oxygen sensors varied slightly in their *in vitro* sensitivity (1 nA = 0.55–0.98 μM) and showed different basal currents during *in vivo* recordings (11–74 nA; mean 26.49 ± 2.04 nA), basal currents were transformed into μM concentrations based on the oxygen sensitivity of each sensor previously determined by the sensor manufacturer. Following this current-concentration transformation, we estimated that basal extracellular levels of oxygen in the NAc in awake, freely moving rats vary within a 7–57 μM range, with a mean of 19.66 ± 1.62 μM (SD = 11.26 μM; *n* = 48). Furthermore, polyphenol-coated sensors showed a slightly lower sensitivity to oxygen *in vitro* (1 nA = 0.75 μM) than bare-wire sensors (1 nA = 0.66 μM), and basal oxygen values detected by these sensors *in vivo* were slightly lower (15.6 μM; *n* = 21) than those detected by bare sensors (29.7 μM; *n* = 12). These estimates of basal oxygen levels are slightly lower than concentrations previously reported in the striatum of freely moving rats (37 ± 16 μM; Bazzu et al., 2009; 33 ± 14 μM; Calia et al., 2009). Further, our μM estimates of basal oxygen concentrations correspond to 6–26 mm Hg of oxygen partial pressure (mean ~13 mm Hg), which is also slightly lower but close to the values reported in awake, resting mice (23 mm Hg; Lyons et al., 2016) and rats (25 mm Hg; Ortiz-Prado et al., 2010) and in lightly anesthetized cats (13 mm Hg; Cross and Silver, 1962).

### Increases in NAc Oxygen Levels Induced by Arousing Stimuli

**Figure 1:**
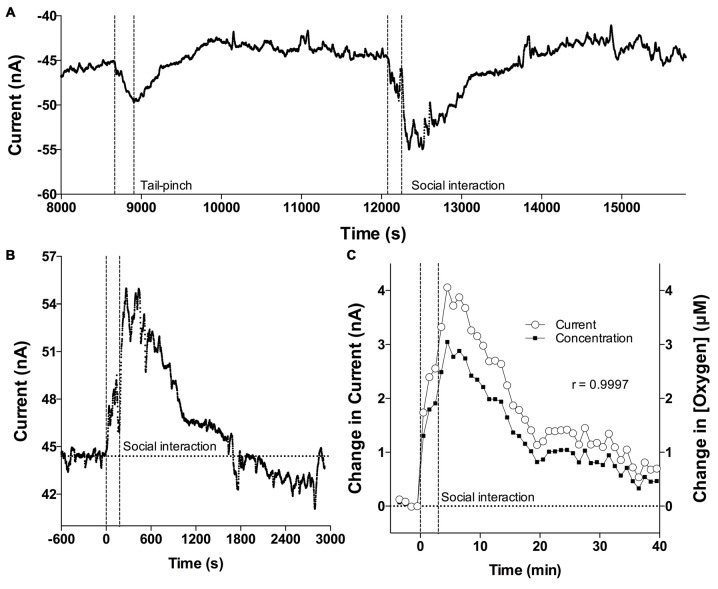
**Oxygen current recording and correlation of oxygen currents and oxygen concentrations. (A)** Original example of changes in reduction current induced by tail-pinch and social interaction. **(B)** Example of high resolution changes in oxygen current induced by social interaction and assessed with 1-s time resolution. **(C)** Mean changes in oxygen induced by social interaction assessed as current and concentration. Both assessments are highly correlative. Vertical hatched lines show onset and offset of stimuli.

Figure 1A shows a representative recording of electrochemical current changes detected in the NAc by an oxygen sensor in an awake, freely moving rat. During a ~3.5-h recording interval, the rat was exposed to two arousing stimuli, a tail-pinch and social interaction. As shown, the original reduction currents are negative, with their decreases reflecting increases in oxygen and *vice versa*. After inversion of these data, changes in currents directly reflect changes in oxygen levels: their increases correspond to oxygen increases and decreases correspond to oxygen decreases. An example in Figure 1B shows a representative recording of changes in oxygen current induced by social interaction sampled with the original 1-s time resolution. Importantly, current and concentration data analyzed with 1-min resolution are virtually identical in their time-course, showing exceptionally strong linear correlation (*r* = 0.999; Figure 1C). Therefore, all subsequent data were expressed in μM concentration units.

**Figure 2:**
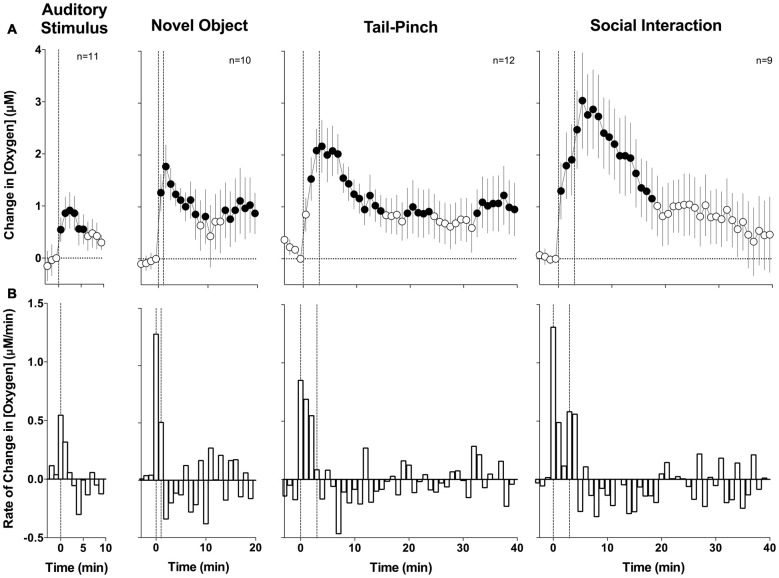
**Mean (±SE) changes in Nucleus accumbens (NAc) oxygen levels induced by arousing stimuli: slow time course analysis.** **(A)** Shows mean changes (1-min bins) and **(B)** shows minute-to-minute rate of changes. Filled symbols show values significantly different from baseline as determined by one-way analyses of variance (ANOVA; *p* < 0.05). Vertical hatched lines show onset and offset of stimuli.

**Figure 3:**
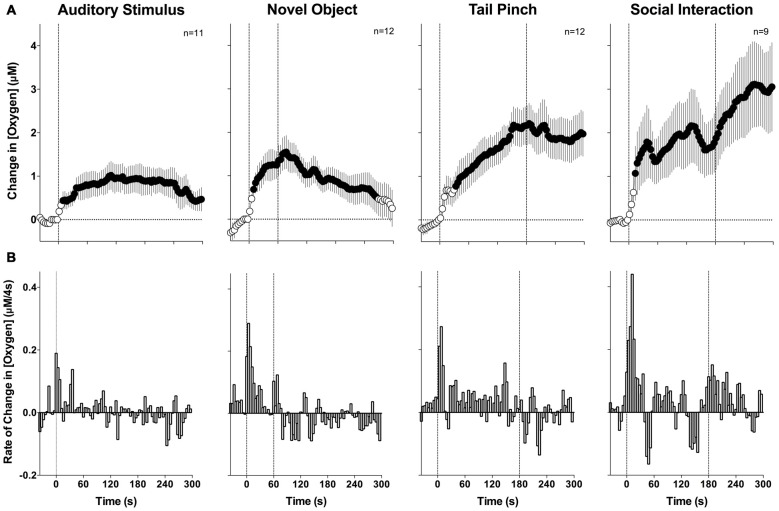
**Mean (±SE) changes in NAc oxygen levels induced by arousing stimuli: rapid time course analysis.** **(A)** Shows mean changes (4-s bins) and **(B)** shows rate of changes. Filled symbols show values significantly different from baseline as determined by one-way ANOVA (*p* < 0.05). Vertical hatched lines show onset and offset of stimuli.

To evaluate changes in NAc oxygen induced by arousing stimuli, two types of analyses were performed. First, we averaged the raw data with slow, 1-min time resolution, transformed current values into concentration, and then expressed them as relative concentration change with respect to the pre-stimulus baseline (Figure 2). Second, since oxygen currents showed rapid changes, the initial periods following stimulus presentation were averaged with rapid, 4-s time resolution to analyze these phasic dynamics (Figure 3).

With slow temporal resolution analysis (Figure 2A), we found that all arousing stimuli significantly increase NAc oxygen levels. The increase was weakest in amplitude and duration after auditory stimulation (*F*_(10,100)_ = 2.51, *p* < 0.01), moderate after novel object presentation (*F*_(9,180)_ = 1.94, *p* < 0.05), and greatest and most prolonged during tail-pinch and social interaction (*F*_(11,440)_ = 2.56 and *F*_(8,320)_ = 3.94, respectively; *p* < 0.01 for both). While the increase in oxygen levels was significant in each group, individual changes were variable, especially at the later time points following stimulus onset. Furthermore, these increases were relatively small when calculated as a percent change relative to the resting oxygen baseline (4.5%, 8.7%, 11.1% and 15.2% for auditory stimulus, novel object presentation, tail-pinch and social interaction, respectively).

Changes in oxygen levels in response to each stimulus were rapid, with the maximal rate of increase occurring during the first minute after the stimuli onset (Figure 2B). These changes also generally matched the duration of stimulation, peaking at the second minute after the beginning of the 1-min novel object presentation and at the fourth minute after the beginning of the 3-min tail-pinch stimulus. Social interaction induced the most prolonged changes and a secondary oxygen increase after the guest rat was removed from the chamber.

Since the most robust changes induced by arousing stimuli occurred during the first minute after stimulus onset, these initial time intervals were analyzed with high temporal resolution (Figure 3). Similar to the slow-resolution analysis, oxygen levels significantly increased after each stimulus (audio *F*_(10,750)_ = 2.05, novel object *F*_(11,825)_ = 3.75; tail-pinch *F*_(11,825)_ = 4.72; social interaction *F*_(8,600)_ = 5.08; for each *p* < 0.01). In each case, the most rapid increase occurred after the stimulus onset and the latency to increase as evaluated with ANOVA with variable time-scale was at the third data point, corresponding to 8–12 s after stimulus onset. In contrast to data analyzed with low resolution, high resolution analysis revealed that the oxygen increase is distinct for each stimulus. The increase was weakest and monotonic after auditory stimulation, whereas the presentation of a novel object resulted in a two-phasic oxygen increase, with the second rise occurring immediately after the novel object was removed from the cage. Tail-pinch induced a continuous increase in oxygen levels that began from the onset of stimulation, continued while the rat actively bit or chewed on the clothespin, and slowly returned to baseline when the clothespin was removed from the rat’s tail. The fastest and largest oxygen increase occurred at the start of and during social interaction. In this case, oxygen levels also displayed a second, rapid but much smaller increase following guest rat removal and corresponding to increased cage exploration by the recorded rat. The response peak occurred at ~5 min from the start and ~2 min after the end of the social interaction stimulus (see Figure 3).

### Possible Mechanisms Underlying Fluctuations in NAc Oxygen Induced by Arousing Stimuli

It is well established that dorsal and ventral striatal neurons have low impulse activity in awake rats during quiet rest but are phasically excited following exposure to various arousing stimuli (Carelli and West, 1991; Kiyatkin and Rebec, 1996, 1999; Rebec, 2006). These neuronal excitations via a neuro-vascular coupling mechanism (local vasodilation followed by rapid rise in local CBF) appear to be the primary cause of phasic increases in NAc glucose levels induced by arousing stimuli (Kiyatkin and Lenoir, 2012). This mechanism was confirmed by microinjecting GLU near the glucose-detecting site in the NAc. GLU injections induced dose-dependent increases in NAc glucose levels presumably due to direct stimulation and excitation of accumbal neurons. To test whether a similar mechanism is involved in rapid increases in NAc oxygen levels induced by arousing stimuli, we used a similar strategy to examine changes in NAc oxygen levels following local intra-NAc GLU injections.

**Figure 4:**
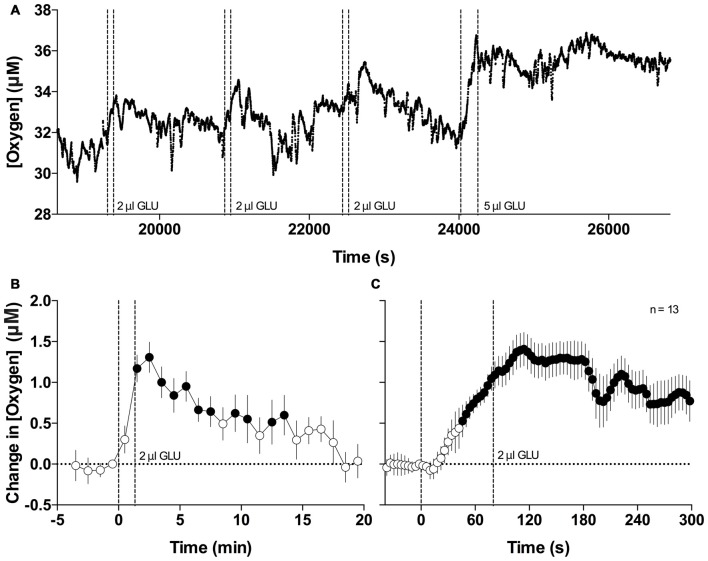
**Changes in NAc oxygen induced by intra-NAc glutamate (GLU) microinjections.** **(A)** Original example of changes in NAc oxygen following repeated microinjections of GLU. **(B)** Mean (±SE) changes in NAc oxygen levels induced by GLU microinjections at 2 μl dose assessed at 1-min time resolution. **(C)** Mean (±SE) changes in NAc oxygen levels induced by GLU microinjections at 2 μl dose assessed at 4-s time resolution. Filled symbols show values significantly different from baseline as determined by one-way ANOVA. Vertical hatched lines show onset and offset of GLU injection.

Figure 4 shows the changes in NAc oxygen elicited by GLU microinjections (**A**, a representative record of oxygen currents following repeated GLU microinjections at different doses; **B** and **C**, mean changes in NAc oxygen induced by GLU at 2 μl dose analyzed with slow and rapid temporal resolution). Local application of GLU consistently increased oxygen levels in each of the five recordings we performed. When injected at the optimal concentration (2 μl of 5 mM solution microinjected over 80 s), the increases were transient, exhibited variable latencies, and displayed relatively low magnitudes (0.5–2 μM) that were similar or lower than those seen with natural arousing stimuli. Since the injecting needle contained saline, the oxygen increases occurred after the second or third GLU application and they usually resulted in slight increases in the oxygen baseline that could be explained by continuous GLU diffusion within the oxygen recording site. When analyzed as a mean of all GLU injections at the same dose, the oxygen increase was highly significant (*F*_(12,240)_ = 4.13 and *F*_(12,900)_ = 5.33 for slow- and rapid time resolutions; *p* < 0.0001), it appeared with ~40-s latency after injection onset, and peaked 30–40 s after injection offset. NAc oxygen responses induced by GLU microinjections were more variable than the previously reported NAc glucose responses, occurred at larger GLU doses, and were typically of lower magnitude (~5%–8% vs. 20%–30%).

Although the results of this experiment suggest that local neuronal activation can increase oxygen entry into the brain tissue, the relatively weak effect incited us to examine another possible mechanism, changes in blood oxygen levels, as a contributor to the increases in NAc oxygen levels induced by arousing stimuli.

An optimal approach to test how blood oxygen content contributes to fluctuations in NAc oxygen is to monitor oxygen levels directly in arterial blood. This approach, however, is technically not feasible in freely moving rats. Therefore, we sought to overcome this obstacle by using an indirect but still reliable approach. Considering that tissue oxygen levels depend on two opposing influences, oxygen inflow from arterial blood and oxygen consumption during metabolic activation, we conducted electrochemical measurements in the subcutaneous space—a densely vascularized area with limited or very low levels of metabolic activity. Our expectation was that the changes in oxygen current recorded from this location would reflect, with a possible latency, changes in oxygen occurring in arterial blood.

**Figure 5:**
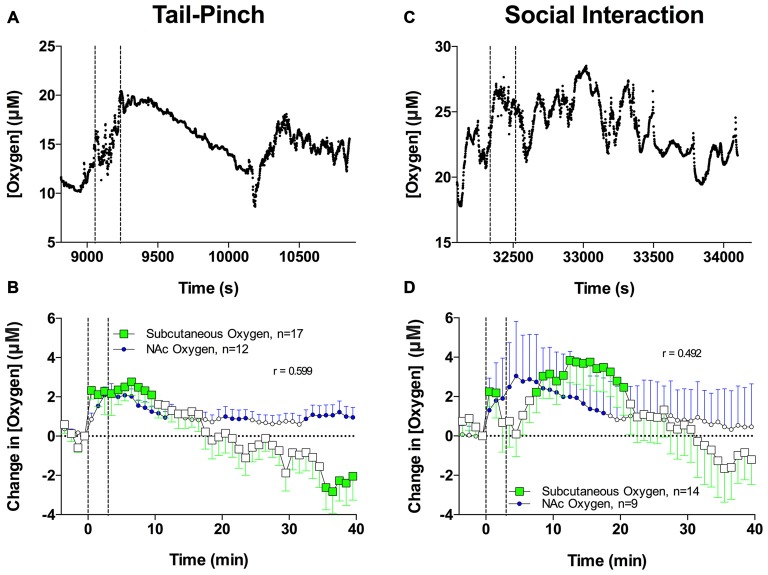
**Brain and skin oxygen changes induced by tail-pinch and social interaction.** **(A)** Individual examples of changes in oxygen in the subcutaneous space induced by tail-pinch. **(B)** Comparison of mean (±SE) changes in NAc and subcutaneous oxygen induced by tail-pinch. **(C)** Individual examples of changes in oxygen in the subcutaneous space induced by social interaction. **(D)** Comparison of mean (±SE) changes in NAc and subcutaneous oxygen induced by social interaction. Filled symbols show values significantly different from baseline as determined by one-way ANOVA. Vertical hatched lines show onset and offset of stimuli.

Figure 5 shows that the tail-pinch and social interaction stimuli significantly increase subcutaneous oxygen levels (*F*_(16,640)_ = 5.79 and *F*_(13,520)_ = 5.85, respectively; both *p* < 0.001). Changes in subcutaneous oxygen induced by stimuli generally paralleled changes in NAc oxygen and displayed significant correlation (*r* = 0.600, 0.492 and 0.675, respectively). Increases in subcutaneous oxygen levels induced by both tail-pinch and social interaction were more variable than increases in NAc oxygen induced by the same stimuli but were similar in magnitude and duration. While basal levels of subcutaneous oxygen varied in different recordings from 8 μM to 50 μM, their mean value (25.36 ± 2.04 μM) was significantly larger (*p* < 0.05) than that observed in the NAc.

### Relationships between Physiological Fluctuations in NAc Oxygen and Glucose

Similar to oxygen, glucose enters brain tissue from arterial blood by gradient-dependent diffusion via GLUT-1 transporters (Duelli and Kuschinsky, 2001). However, in contrast to oxygen, for which blood levels are variable and dependent on respiration, glucose is normally present in the blood at relatively stable levels. Enhanced entry of glucose into brain tissue occurs due to proximal neuronal activation, which induces local vasodilation and increases CBF (Fellows et al., 1992; Silver and Erecińska, 1994; Attwell et al., 2010; Mergenthaler et al., 2013). If the entry of oxygen and glucose into the brain is governed by identical or similar mechanisms, we hypothesized that the patterns of change in glucose and oxygen would share important similarities. To test this hypothesis, we compared our current oxygen data with previously reported data on changes in NAc glucose induced by the same arousing stimuli (Kiyatkin and Lenoir, 2012; Wakabayashi and Kiyatkin, 2015).

**Figure 6:**
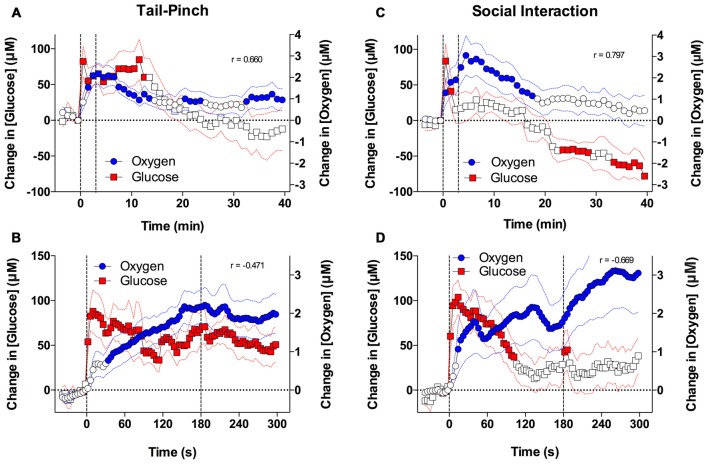
**Brain oxygen and glucose dynamics induced by tail-pinch and social interaction.** **(A)** Comparison of mean (±SE) changes in NAc oxygen and glucose concentrations induced by tail-pinch and analyzed at low (1-min) time resolution. **(B)** Comparison of mean changes in NAc oxygen and glucose concentrations induced by tail-pinch and analyzed at high (4-s) time resolution. **(C)** Comparison of mean changes in NAc oxygen and glucose concentrations induced by social interaction and analyzed at low time resolution. **(D)** Comparison of mean changes in NAc oxygen and glucose concentrations induced by social interaction and analyzed at high time resolution. Filled symbols show values significantly different from baseline as determined by one-way ANOVA. Vertical hatched lines show onset and offset of stimuli.

From this comparison, we found that basal levels of glucose in the NAc (~500 μM) were much larger than those for dissolved oxygen (~20 μM). Similarly, the absolute magnitude of glucose response induced by arousing stimuli was proportionally larger in terms of concentration than for oxygen. However, as a percent change relative to the baseline, the response magnitude was comparable for both parameters (10%–20%). Similar to oxygen, both tail-pinch and social interaction increased NAc glucose levels (Figures 6A,C). When the data were quantified with slow, 1-min time resolution, the changes in oxygen and glucose significantly correlated during exposure to both stimuli. However, glucose levels displayed maximal increase and peaked during the first minute of stimulation, whereas oxygen levels increased more tonically and peaked after the end of the tail-pinch and social interaction stimuli.

Drastic differences in the time-course of stimulus-induced fluctuations in glucose and oxygen were evident when the data were analyzed with high temporal resolution (Figures 6B,D). In contrast to the rapid, accelerating rise of oxygen, glucose levels peaked phasically at ~10 s after the start of the tail-pinch and social interaction stimuli and then slowly returned to the pre-stimulus baseline. Thus, the increases in NAc glucose were clearly more phasic and more transient than the oxygen increases. Due to these differences in the rapid time-course, changes in oxygen and glucose negatively correlated; when glucose started to decrease after the initial peak, oxygen continued to rise.

### Relationships between Changes in NAc Oxygen and Temperature Parameters

As shown in our previous studies (for review see Kiyatkin, 2010), any arousing stimulus presented to awake rats elicits moderate increases in brain temperature that depend on two primary mechanisms: (1) an increase of intra-cerebral heat production due to metabolic brain activation; and (2) a decrease in heat loss to the external environment due to peripheral vasoconstriction. Since metabolic brain activation increases intra-cerebral heat production (Hodgkin, 1967; Ritchie, 1973; Siesjo, 1978; Sokoloff, 1999), we examined the relationship between changes in NAc oxygen and different temperature parameters induced by two distinct arousing stimuli, tail-pinch and social interaction.

**Figure 7:**
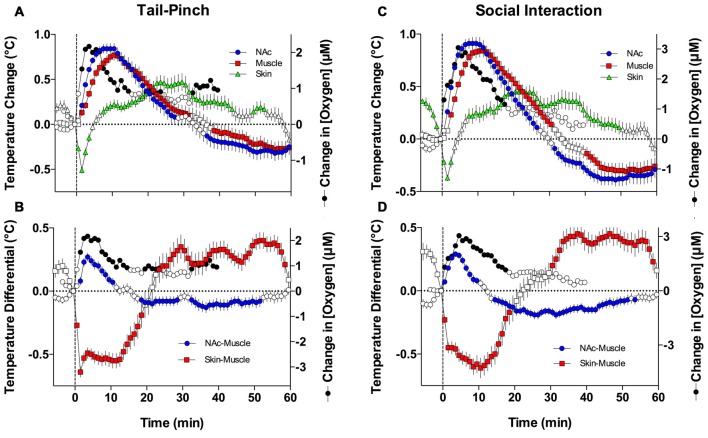
**Oxygen dynamics and changes in temperature parameters during tail-pinch and social interaction. (A)** Comparison of mean (±SE) changes in NAc oxygen and NAc, muscle, and skin temperatures induced by tail-pinch. **(B)** Relationships between oxygen and NAc-muscle and skin-muscle temperature differentials following tail-pinch. **(C)** Comparison of changes in NAc oxygen and NAc, muscle, and skin temperatures induced by social interaction. **(D)** Relationships between oxygen and NAc-muscle and skin-muscle temperature differentials following social interaction. Filled symbols for temperature parameters shown values significantly different from baseline as determined by one-way ANOVA. Oxygen values are shown without standard errors for clarity.

**Figure 8:**
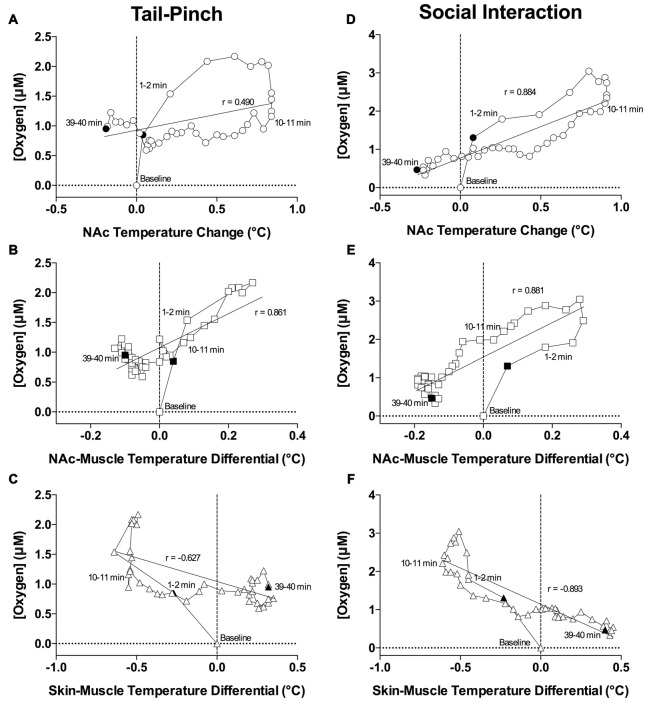
**Correlative relationships between changes in NAc oxygen and temperature parameters. (A)** Correlation between oxygen concentration and NAc temperature change induced by tail-pinch. **(B)** Correlation between oxygen concentration and NAc-muscle differential induced by tail-pinch. **(C)** Correlation between oxygen concentration and skin-muscle temperature differential induced by tail-pinch. **(D)** Correlation between oxygen concentration and NAc temperature change induced by social interaction. **(E)** Correlation between oxygen concentration and NAc-muscle differential induced by social interaction. **(F)** Correlation between oxygen concentration and skin-muscle temperature differential induced by social interaction. Filled symbols represent the bounds of data included in the correlation analysis.

As shown in Figures 7A,C, both oxygen levels and temperature recorded from the NAc increased during tail-pinch and social interaction; these parameters significantly correlated for both stimuli, but the strength of correlation was larger for social interaction (*r* = 0.884, *p* < 0.0001) than for tail-pinch (*r* = 0.590, *p* < 0.01). However, oxygen increases occurred more rapidly, more strongly, and peaked at an earlier time than changes in brain temperature (Figures 8A,D). In each case, oxygen levels surged strongly during the first minute of stimulation, when brain temperature had only just begun to increase and was still close to baseline. For tail-pinch, oxygen levels continued to increase during the next 2–3 min and showed a strong direct correlation with brain temperature. This correlation disappeared from ~8 min after stimulus onset, when both parameters began to decrease toward baseline. Although oxygen levels also jumped rapidly during the first minute of social interaction preceding any real change in brain temperature, the correlation between the two parameters (*r* = 0.884) was stronger than for tail-pinch for the entire 40-min analysis interval.

Since the temporal muscle is not involved in locomotion and receives its arterial blood supply from the carotid artery like the brain does, the difference between brain and muscle temperature (or brain-muscle differential) eliminates the influence of arterial blood temperature on the brain, thus allowing us to reveal a component of the temperature response that is related to intra-brain heat production resulting from metabolic brain activation (Kiyatkin, 2010). This parameter consistently increased following exposure to various arousing stimuli, showing an apparent correlation with their arousing potential. As shown in Figures 7B,D and 8B,E, oxygen levels displayed a tight direct correlation with brain-muscle differentials (*r* = 0.861 and = 0.881, *p* < 0.0001 for tail-pinch and social interaction, respectively) for the entire analysis interval apart from the short time intervals following the stimulus onset. During this initial, ~1-min time interval following the onset of tail-pinch and social interaction, oxygen levels rapidly and strongly increased preceding weaker increases in NAc-muscle temperature differentials.

Although skin temperature is primarily dependent on vascular tone, typically displaying a biphasic, down-up fluctuation during the arousing stimulation, the skin temperature response is also affected by the temperature of arterial blood. The skin-muscle differential eliminates this influence and provides an accurate index of skin vascular tone (vasoconstriction/vasodilation). As shown in Figures 7B,D, oxygen levels also significantly correlated with skin-muscle differentials, but the correlation was negative in both cases and quantitatively stronger for the social interaction stimulus (*r* = −0.893) than for the tail-pinch stimulus (*r* = −0.627; Figures 8C,F). Both parameters changed very rapidly, reaching nadir within 4–6 min.

## Discussion

Although the first electrochemical sensors for *in vivo* monitoring of dissolved oxygen were introduced long ago (Davies and Brink, 1942; Clark et al., 1958; Kahn, 1964), our basic understanding about how oxygen enters and fluctuates in the brain tissue under normal physiological and behavioral conditions remains unclear. Therefore, our primary goal was to describe physiological fluctuations in brain oxygen levels and explore their underlying mechanisms using high-speed electrochemical monitoring in freely moving rats. This study is a logical extension of our previous work, in which we assessed physiological fluctuations in brain glucose levels using enzyme-based biosensors and a similar experimental protocol (Kiyatkin and Lenoir, 2012). Comparing these data allowed us to examine the similarities and differences in how these two critical metabolic substances change in the brain in response to various arousing stimuli. Finally, we used data from our previous thermorecording studies (Kiyatkin, 2010) to explore how changes in brain oxygen and glucose levels are related to metabolic brain activation assessed by intra-cerebral heat production.

### Electrochemical Evaluations of Brain Oxygen Dynamics: Methodological Considerations

In this study, constant potential amperometry with Pt-Ir oxygen sensors was employed to evaluate fluctuations in oxygen levels in the NAc of an awake, freely moving rats. Although measurement selectivity is a critical issue in any electrochemical study, oxygen differs from most brain neurochemicals because it is reduced, not oxidized, by the applied voltage. Therefore, changes in reduction currents detected *in vivo* appear to be free from contamination by the multiple oxidizable substances contained in the brain’s extracellular space. In contrast to the relatively easy *in vitro* testing of carbon-fiber and enzyme-based sensors for their substrate sensitivity, selectivity, and temperature dependence, these tests for oxygen sensors are more technically complex and sometimes even impossible. For example, the strategy of enzyme-free or “null” sensor recordings, an important approach to control for possible physical and chemical contaminants to neurochemical-specific currents detected by enzyme-based sensors (Kiyatkin and Lenoir, 2012; Kiyatkin and Wakabayashi, 2015), is impossible with oxygen sensors. For current-to-concentration conversion, we relied on oxygen sensitivity values carefully determined *in vitro* for each sensor at 37°C by the sensors’ manufacturer. We also relied on previous data, which demonstrates that Pt oxygen sensors of similar design are fully insensitive to physiological levels of most oxidizable substances, including ascorbic acid, uric acid, dopamine and its primary metabolites (Bolger et al., 2011). However, the true sensitivity and selectivity of electrochemical sensors in real brain environment differ from their *in vitro* values, possibly affecting some quantitative values for oxygen baseline and oxygen responses to stimuli in this study. Particularly, basal levels of oxygen could be slightly undervalued because we were unable to perform post-recording calibrations and used for calculations the pre-recording calibration data.

While many previous oxygen studies were performed in anesthetized animals (for review see Masamoto and Tanishita, 2009), our recordings were conducted in awake, freely-moving rats. Given that general anesthesia causes metabolic inhibition, profound brain hypothermia, and has dramatic effects on the tone of cerebral vessels (Kiyatkin and Brown, 2005), our study represents a more accurate estimate of brain oxygen dynamics under physiologically relevant conditions. These effects of anesthesia have been shown to seriously distort the entry of oxygen and glucose into the brain and their basal levels in the brain tissue. For example, while natural arousing stimuli generate 5%–20% increases in NAc glucose (Kiyatkin and Lenoir, 2012; see Figure 6), general anesthesia induced by a pentobarbital-chloral hydrate mixture almost doubles glucose levels in this structure, presumably via dilation of cerebral vessels (Bola and Kiyatkin, 2016). In contrast to strong, non-physiological stimuli and animal exposure to air with dramatically different oxygen content often used in oxygen studies focused on brain hypoxia, our rats were exposed to natural arousing stimuli of different nature. The effects of these stimuli have been previously assessed on behavioral, physiological, neuronal, and neurochemical levels that allowed us to consider a local oxygen response in relation to changes in other related parameters. Finally, our recordings were conducted with high temporal resolution, which allowed us to reveal rapid oxygen fluctuations that are easily missed when the data are analyzed with a coarser temporal resolution.

### Mechanisms Underlying Physiological Increases in NAc Oxygen Levels

The primary finding of this study is that NAc oxygen levels increase significantly following exposure to various arousing stimuli ranging from a weak stimulus, such as an auditory tone, to a strong, aversive stimulus, such as a tail-pinch. The increases are rapid, appearing within several seconds from the stimulus onset (see Figure 3) and their magnitude (+5–20%) and duration (5–20 min) paralleled the changes in locomotor activity and other physiological parameters (Kiyatkin et al., 2002; Kiyatkin, 2010). As previously described, all stimuli used in this study phasically excite accumbal neurons (Kiyatkin and Rebec, 1996, 1999; Kiyatkin and Brown, 2007) due to phasic GLU release (Kiyatkin and Rebec, 1999; Wakabayashi and Kiyatkin, 2015) and increase intra-brain heat production that suggests metabolic brain activation (Kiyatkin et al., 2002; Kiyatkin and Smirnov, 2010; see Figure 7 in this study). Since oxygen levels reflect a balance between two opposing forces: oxygen inflow from the arterial blood and oxygen consumption due to metabolic activity, it is reasonable to assume that *increases* in NAc oxygen result from enhanced oxygen entry into the extracellular space.

Considering the possible mechanisms responsible for the increased inflow of oxygen elicited by arousing stimuli, we first focused on neuro-vascular coupling, which postulates that the entry of oxygen into the brain tissue is triggered by neuronal activation that induces local vasodilation and an increase in local CBF (Fox and Raichle, 1986; Fellows and Boutelle, 1993; Attwell et al., 2010). As shown in our experiment with intra-NAc GLU injections, this neural mechanism is indeed involved in the phasic increases in NAc oxygen levels (see Figure 4). However, in contrast to the rapid, consistent, and dose-dependent increases in NAc glucose induced by local GLU applications in our previous study (Kiyatkin and Lenoir, 2012), GLU-induced increases in NAc oxygen are more variable and relatively weak even at higher GLU doses. Therefore, although local neuronal activation can trigger a chain of events resulting in increased CBF and although this mechanism is certainly involved in mediating oxygen increases induced by arousing stimuli, our data indicate that other factors should be considered. These include the direct influences on the cerebral vessels from other neural structures, an increase in blood inflow to the brain caused by peripheral vasoconstriction and between-structure redistribution of blood (Roy and Sherrington, 1890), and the increase in perfusion pressure due to the rise in arterial pressure, heart rate, and cardiac output—the basic physiological effects elicited by arousing stimuli.

Increases in NAc oxygen induced by arousing stimuli could also be the result of an increase in blood oxygen levels due to increased respiration. Classic physiological studies demonstrate that arousing and stressful stimuli, including those used in this study, strongly increase respiration rate, the volume of air inspired, and brain oxygen uptake (Bedford et al., 1979; Kabir et al., 2010) resulting in increased saturation of hemoglobin and greater oxygen diffusion into brain tissue from dilated cerebral vessels. Since direct monitoring of blood oxygen is technically complex and remains impossible in freely moving rats, the subcutaneous recording site was useful for our purposes because oxygen values detected from this location should reflect blood oxygen levels outside of the influence of oxygen consumption by organ tissue.

Both tail-pinch and social interaction significantly increase subcutaneous oxygen levels, which suggests that increased oxygenation of arterial blood also contributes to the increases in NAc oxygen levels. While increased blood flow in the subcutaneous space could alternatively explain this phenomenon, this is unlikely because tail-pinch and social interaction induce phasic decreases in skin temperature (see Figure 7A), linked to peripheral vasoconstriction and decreased oxygen availability. We also found that basal oxygen levels in the subcutaneous space were slightly but significantly larger than those in the NAc, but stimuli-induced increases were similar in both locations.

Finally, all arousing stimuli variably increase brain temperature (see Figure 7). Since increases in temperature facilitate the diffusion of gases in liquids (first Fick’s law; Longsworth, 1954; Han and Bartels, 1996), stimuli-induced elevations in brain temperature should enhance oxygen diffusion into brain tissue, thus contributing to increases in NAc oxygen. Although NAc oxygen increases induced by tail-pinch and social interaction generally paralleled changes in brain temperature and showed moderate correlations (Figure 7), changes in temperature and oxygen were distinct, both directly following and well after stimulus presentation (see Figure 8).

### Relationships between Brain Changes in Oxygen and Glucose and Metabolic Brain Activation

While oxygen and glucose both enter the brain from arterial blood via a concentration-dependent mechanism, the dynamics of their physiological fluctuations assessed in the NAc have similarities and important differences (see Figure 6). Despite profound differences in basal concentrations, increases in NAc oxygen and glucose elicited by tail-pinch and social interaction were of similar relative magnitude (5%–20% above baseline) and duration (10–20 min). In each case, the increases occurred with surprisingly short and similar latencies (4–8 s), suggesting the involvement of a common neural mechanism, which was confirmed by using intra-NAc GLU microinjections. Finally, rapid time-course analyses revealed that increases in both glucose and oxygen precede the increases in brain-muscle temperature differentials, which results from intra-brain heat production due to metabolic brain activation. Therefore, due to neural activation and the subsequent rapid rise in local CBF induced by arousing stimuli, the brain receives more oxygen and glucose in advance of its metabolic demand, thus preventing potential metabolic deficits.

Although neuro-vascular coupling could explain the equally short response latencies, the increase in glucose was more phasic and transient than the oxygen increase. The greatest change in NAc glucose was observed at the start of the tail-pinch and social interaction, whereas the oxygen response was more tonic, multiphasic, and prolonged. Therefore, these data are consistent with previous studies (Attwell et al., 2010), suggesting that neuro-vascular coupling appears to be the primary mechanism regulating glucose entry into the brain during functional neural activation. In contrast, oxygen entry into the brain appears regulated by multiple, time-dependent mechanisms.

### Conclusions and Functional Implications

The present study reveals that extracellular oxygen levels in the NAc in awake, freely moving rats are not stable, showing rapid (4–10 s latencies), relatively modest (5%–15% above baseline), multiphasic increases following exposure to natural arousing stimuli. We also clarified that these increases result from enhanced oxygen entry from arterial blood that is mediated via several time-dependent mechanisms. While we confirmed that local neural activation followed by a rapid vasodilatory response (neuro-vascular coupling) is a primary factor increasing oxygen inflow into brain tissue, this inflow is enhanced by slower elevations in blood oxygen levels due to respiratory activation induced by arousing stimuli. Natural arousing stimuli also modestly increased NAc glucose levels (5%–20%); these increases were equally rapid but more phasic and transient than oxygen increases. Changes in oxygen and glucose showed similar dynamics at the initial time intervals following stimuli onset, suggesting neuro-vascular coupling as a common mechanism enhancing brain entry of both substances. This neural mechanism appears to be primary in providing rapid entry of glucose and phasically increasing its levels following exposure to arousing stimuli. While blood glucose levels are strictly regulated and maintained at relatively stable levels within the behavioral continuum (Gerich, 1993), they can strongly increase following the consumption of glucose-containing products, resulting in a rise in brain glucose levels (Wakabayashi and Kiyatkin, 2015). Therefore, the gradient-dependent mechanism appears to be responsible for robust glucose entry into the brain tissue and the strong tonic increases in glucose levels observed in the context of feeding behavior and food consumption (Wakabayashi et al., 2016). In contrast to glucose, brain oxygen levels have relatively strict upper limits, exhibiting relatively weak increases following arousing stimulation. However, due to strong dependence on respiration, brain oxygen levels could dramatically fall following respiratory depression due to a reduction in blood oxygen levels. Respiratory depression is out of normal physiological domain but it is well described during anesthesia and opioid drug use (Yeadon and Kitchen, 1990; Jaffe et al., 1997; Stohler et al., 1999; Dursteler-Mac Farland et al., 2000). Investigating respiratory depression in the context of anesthesia and opioid drugs may be a fruitful avenue for future research.

When analyzed with slow temporal resolution, changes in oxygen significantly correlated with changes in brain temperature and intra-brain heat production, suggesting their general association. However, rapid time-course resolution analyses revealed that oxygen and glucose began to increase within several seconds after stimuli onset that preceded any changes in brain metabolic activity. Therefore, by perceiving environmental stimuli, inducing neural activation and triggering a chain of neuro-vascular events, during functional neural activation the brain receives more oxygen and glucose in advance of its metabolic need, thus preventing possible metabolic deficits. This “anticipatory” mechanism is highly effective for providing the proper energetic recourses to sustain a wide range of brain functions spanning the physiological and behavioral continuum, but its efficiency could be seriously compromised by neuroactive drugs that affect brain metabolism and the tone of cerebral vessels.

## Author Contributions

EAK: concept and design; ES and EAK: research performance; ES, KTC-B and EAK: data analyses; EAK: primarily responsible for writing the manuscript.

## Funding

This research was supported by the National Institute on Drug Abuse—Intramural Research Program, NIH (1ZIADA000566-05).

## Conflict of Interest Statement

The authors declare that the research was conducted in the absence of any commercial or financial relationships that could be construed as a potential conflict of interest.
